# Mr. Low, on Ring-Worm of the Head

**Published:** 1805-01-01

**Authors:** Robert Low

**Affiliations:** Member of the Royal College of Surgeons. Tavistock Row, Covent Garden


### *To the Editors of the Medical and Physical Journal.*

GENTLEMEN,

I Flatter myself, that amongst the extensive circle of Readers of your very valuable miscellany, I shall find some practitioners of observation and experience, who will endeavour to investigate more satisfactorily than has hitherto been done, the origin, nature, and cure of a disease, which appears to me highly to merit the attention of the profession; not only because it has already excited very little of the attention of medical writers, but also, because its occurrence appears to be daily becoming more and more common. The disease to which I here allude is best known by the appellation of "The Ring-Worm in the Head."

It is a fact, I believe, not generally known, that this disorder is more dreaded in the schools about London, than any other epidemic; its origin, natural mode of communication, and cure, seem all to be involved in equal uncertainty.

The general opinion of those, who have endeavoured to find out the antiquity of this disorder, is, that it was imported from India, not more than twenty or thirty years ago; and what appears to favour this opinion, is the concurring testimony of schoolmasters, that Indian boys are more subject to it than the natives of this island; but whether it is merely a local disease, or whether it depends upon a disordered state of the whole system, so as to require internal medicines for its eradication, is a question which has not yet been decided; neither has it been ascertained whether the infection is communicated by bodily contact, or by the inhalation of air which has been respired by the disordered person, or by cutaneous absorption tion, &c. These are points, which I mention not with a view of being able to clear them of their difficulties, by any observations which I have myself made, or any information which I have received; but because, by a public notice of them, I may induce some persons of superior talents and greater experience, to prosecute that inquiry which may ultimately benefit society, and enrich the present stock of medical knowledge. I shall, however, take the liberty of describing the "Ring-Worm of the Head," in its different stages, as clearly as I have been able to ascertain them, and then enumerate the different applications which have been put in practice, making such remarks as may tend to fix their comparative merits, provided the enumeration of *inefficacious* applications may be considered as steps towards the discovery of efficacious remedies.

I need not, it is presumed, premise any thing respecting the nature of a *common* ring-worm, which different parts of the body are liable to, for it will generally yield to any of the common astringent applications to the affected surface; but inasmuch as a ring-worm of the *hairy scalp* will not yield to the same treatment with that on other parts of the body, may it be considered as a different species of the same genus?

Most usually the first appearance of a "ring-worm of the head," is that of a small circle of redness, from the size of a half guinea to that of a guinea, and red only at the circumference, which comes on very suddenly, and occasions the hair to separate at the roots from the slightest touch; this circle increases somewhat in diameter by degrees, and contracts a branny scurf, which constitutes the disorder.

It has been asserted, that animalculæ under the cuticle are the cause of this disorder; but a clergyman of my acquaintance, who, from having a numerous and respectable school, is much interested in the discovery, has made very accurate examination with a microscope, and must have detected *motion*, had there been any living animalculæ; but the result was, that no such cause of irritation existed.

When one circle has made its appearance in a child's head, (for I do not find that adults are ever affected with the disease, nor are girls so subject to it as boys) it may be expected that other similar circles will appear in a short time, on different parts of the head, till they reach one to another, and thus gain possession of the whole hairy scalp:

scalp: when the original circle has considerably enlarged itself, and others have broken out, a confusion follows by means of the intersections, and unless the disorder be subdued, glandular swellings will ensue, and sometimes ulcerations.

On inquiring of my friend before alluded to, whether any symptoms ever precede this disorder, which may be considered as indicative of its approach; I was informed that a paleness of countenance, attended with a loss, or at least a diminution of appetite, so frequently gives notice of its approach, that he has many times pronounced his apprehension, that Master *Such-a-One* was going to have a ring-worm, which has shortly afterwards proved to be the case. This prognosis has not, that I know of, been noticed; perhaps not known to any medical writer; and, therefore, deserves to be more minutely attended to by those who undertake to ascertain the class in which this disease ought to be ranked.

In attempting to cure this disorder, not only have different medical men differed widely in their modes of treatment, but the same person has been under the necessity of trying various applications successively, before the end could be accomplished; which empirical kind of treatment has left him in the dark, as to what particular application proved of the greatest service, or whether it was the succession of different applications which jointly effected the cure.

When a practitioner is applied to for curing the disease in question, who makes no distinction between it and a common ring-worm of any other part, he most commonly applies a solution of sulphate of zinc in rose water, which lotion, though applied at a very early period, very seldom stops the progress of the disorder, and, in almost every instance, fails of effecting a cure: after the astringent lotion has been applied for some time, the *ung. picis* is perhaps recurred to, the effects of which appear to be nearly the same as those of the former application. At length, when much time has elapsed in fruitles efforts to cure the disorder, the hair is taken off, and the head shaven, as the "dernier resource," and blisters and plasters of Burgundy pitch have also been applied: this supposes the disease to be merely local; but from what information I can collect, topical applications *alone* have seldom been found sufficiently powerful to effect a radical cure.

It

It would be improper to mention *names* in a paper of this kind, which is principally intended to induce further inquiry into the nature and cure of the "ring-worm of the head;" otherwise I could refer to the practice of a gentleman, who uses *internal* medicines as well as *topical* applications, and, perhaps, with more success than any of his cotemporaries. About five years ago, a school in the confines of London had not less than sixteen young gentlemen infected with this disorder at the same time; in whom, the applications above specified, besides some others not worthy of particular notice, were resorted to without success, but were ultimately cured by the attention of the gentleman just alluded to. The treatment was thus: The young gentlemen had their heads shaven every four or five days, and took powders of Æthiop's mineral, sulphate of kali, and calcined magnesia twice a day, whilst the ung. hyd. nitrat. was daily applied over the bare head at bed-time, and washed off the next morning with a piece of flannel and soap-suds. This treatment rigidly adhered to, the diet in the mean time being light, the disorder was eradicated; and this treatment has in other instances since been found equally successful, though it must be confessed, that three or four months usually elapse before a cure is effected. It may not be amiss here to add, that the school in which this disorder occurred, was remarkable for cleanliness, regular exercise, airy rooms, good bedding, and proper diet; and that there was a limitation in their number of pupils. Other schools have been equally infected with this disorder, inasmuch that the master of one, which I have heard of, not long ago, changed all the bedding, from an idea that the contagion was propagated by it; and another declared to his apothecary, that he had sustained a greater injury from this complaint than would be repaired, to use his own expression, by "a hatful of gold."

If what I have advanced should be deemed destitute of arrangement or inconclusive, let it be considered that the want of sufficient experience in the disorder in question, apologises for my not offering any thing more conclusive at present; but I trust that the candid reader will at least allow me the credit due to my intention of calling forth the attention of men skilful, as well as more experienced heads, to a subject, which, in my opinion, greatly deserves the investigation of medical research.

The concluding remark, which I must not omit to make, is, is, that the growing again of the hairs may be considered as a criterion of approaching health.* I am, &c.

ROBERT LOW,
Member of the Royal College of Surgeons.

*Tavistock Row, Covent Garden, Oct.* 18, 1804.

P. S. I shall, with your permission, avail myself of your next Number, to describe a portable electric machine, very convenient for medical purposes, which was invented some years ago by my brother-in-law, the Rev. William Pearson, and has since been somewhat improved by myself; I intend, also, if I have time, accompanying it with some cases, in which I have applied electricity with great benefit.

* The Editors consider Mr. L's communication as a very important one. They believe the disease to be contagious, and a disease of the hairs, which may be cured by eradicating them. Ed.

